# A petunia transcription factor, *PhOBF1*, regulates flower senescence by modulating gibberellin biosynthesis

**DOI:** 10.1093/hr/uhad022

**Published:** 2023-02-16

**Authors:** Xiaotong Ji, Ziwei Xin, Yanping Yuan, Meiling Wang, Xinyi Lu, Jiaqi Li, Yanlong Zhang, Lixin Niu, Cai-Zhong Jiang, Daoyang Sun

**Affiliations:** College of Landscape Architecture and Arts, Northwest A&F University, Yangling, Shaanxi 712100, China; College of Landscape Architecture and Arts, Northwest A&F University, Yangling, Shaanxi 712100, China; College of Landscape Architecture and Arts, Northwest A&F University, Yangling, Shaanxi 712100, China; College of Landscape Architecture and Arts, Northwest A&F University, Yangling, Shaanxi 712100, China; College of Landscape Architecture and Arts, Northwest A&F University, Yangling, Shaanxi 712100, China; College of Landscape Architecture and Arts, Northwest A&F University, Yangling, Shaanxi 712100, China; College of Landscape Architecture and Arts, Northwest A&F University, Yangling, Shaanxi 712100, China; College of Landscape Architecture and Arts, Northwest A&F University, Yangling, Shaanxi 712100, China; Department of Plant Sciences, University of California, Davis, Davis, CA 95616, USA; Crops Pathology and Genetics Research Unit, USDA-ARS, Davis, CA 95616, USA; College of Landscape Architecture and Arts, Northwest A&F University, Yangling, Shaanxi 712100, China

## Abstract

Flower senescence is commonly enhanced by the endogenous hormone ethylene and suppressed by the gibberellins (GAs) in plants. However, the detailed mechanisms for the antagonism of these hormones during flower senescence remain elusive. In this study, we characterized one up-regulated gene *PhOBF1*, belonging to the basic leucine zipper transcription factor family, in senescing petals of petunia (*Petunia hybrida*). Exogenous treatments with ethylene and GA_3_ provoked a dramatic increase in *PhOBF1* transcripts. Compared with wild-type plants, *PhOBF1*-RNAi transgenic petunia plants exhibited shortened flower longevity, while overexpression of *PhOBF1* resulted in delayed flower senescence*.* Transcript abundances of two senescence-related genes *PhSAG12* and *PhSAG29* were higher in *PhOBF1*-silenced plants but lower in *PhOBF1*-overexpressing plants. Silencing and overexpression of *PhOBF1* affected expression levels of a few genes involved in the GA biosynthesis and signaling pathways, as well as accumulation levels of bioactive GAs GA_1_ and GA_3_. Application of GA_3_ restored the accelerated petal senescence to normal levels in *PhOBF1*-RNAi transgenic petunia lines, and reduced ethylene release and transcription of three ethylene biosynthetic genes *PhACO1*, *PhACS1*, and *PhACS2*. Moreover, PhOBF1 was observed to specifically bind to the *PhGA20ox3* promoter containing a G-box motif. Transient silencing of *PhGA20ox3* in petunia plants through tobacco rattle virus-based virus-induced gene silencing method led to accelerated corolla senescence. Our results suggest that PhOBF1 functions as a negative regulator of ethylene-mediated flower senescence by modulating the GA production.

## Introduction

Senescence is a genetically programmed event that occurs in the terminal phase of individual tissue or organ development in plants. It is typically characterized by wilting, discoloration, and even abscission due to the sequential breakdown of physiological and biochemical activities [1]. Although senescence makes a great contribution to plant survival by allowing nutrient recycling and reallocation [2], it may cause substantially reduced crop yield and biomass production from an agronomic perspective. The retardation of senescence process is essential for many plant species, especially ornamental plants. Numerous studies have been performed to investigate the molecular regulatory mechanisms of senescence in various plant organs, including roots [3, 4], leaves [5, 6], stems [7], fruits [8, 9], and flowers [10, 11]. These studies demonstrate that senescence is a complicated biological process which involves a wide range of degradative, biosynthetic, and regulatory mechanisms coordinated by gene expression.

The initiation of flower senescence is concomitant with significant alterations in the cellular components. Plant hormones are considered to impose important influences on the senescence of floral organs. It has been revealed that ethylene serves as a major regulator of flower senescence in ethylene-sensitive plants, whose senescence process shows increased ethylene release in a climacteric-like pattern [12]. Exogenous treatment with ethylene accelerates petal senescence and promotes the transcription of senescence-associated genes [13]. Abscisic acid (ABA) is also known as a promoter of flower senescence, and its treatment resulted in accelerated senescence of floral organs and deterioration of postharvest quality in miniature potted rose [14]. Particularly, ABA was thought to be a primary contributor to flower senescence in ethylene-insensitive gladiolus [15]. Another hormone, jasmonic acid (JA), was shown to have similar function in accelerating senescence progression of *Dendrobium* orchid flowers [16]. Contrarily to the senescence-promoting effects of the hormones above, cytokinins (CTKs) and gibberellins (GAs) are proposed as important anti-senescence factors in the corollas. It has been indicated that application of GAs extends flower life of a number of plant species, such as iris [17], rose [18], allamanda [19], tobacco [20], and freesia [21].

It is well recognized that the regulation of flower senescence depends greatly on a complex hormonal crosstalk rather than individual hormone. For example, ethylene and ABA have been indicated to interact in senescing rose flowers under water limitations [22]. ABA treatment repressed ethylene production during flower senescence of hibiscus [23], whereas the treatment of carnation flowers with an ethylene action inhibitor, silver thiosulfate, restricted ABA-stimulated senescence process [24]. It has been reported that the interplay between JA and ethylene acts in parallel to regulate the timing of floral organ abscission and senescence [25]. GAs have been shown to be antagonistic to both ethylene and ABA in senescing corollas, and the decline in the accumulation of bioactive GAs promoted ethylene- and ABA-mediated rose petal senescence [18]. A recent study suggested that ethylene treatment reduced the expression of GA biosynthetic genes and increased the transcription of GA catabolic genes in rose flowers [26]. However, how these hormonal interactions are transcriptionally regulated is largely unclear.

The basic leucine zipper (bZIP) transcription factors comprise an extensive and conserved family, which is divided into 10 distinct groups in plants [27]. The group S contains the most bZIP members, which are associated with plant growth and development. Among them, AtbZIP11 from Arabidopsis has been demonstrated to regulate amino acid and sugar metabolism by specifically activating *asparagine synthetase 1* and *proline dehydrogenase 2* [28]. AtbZIP11 has been identified as a quantitative modulator of auxin-stimulated responses through the regulation of histone acetylation [29]. The functions of some AtbZIP11 homologs from other plant species have also been studied. For instance, an up-regulation of *tbz17*, a tobacco gene that is phylogenetically related to *AtbZIP11*, was observed during leaf senescence [30], and constitutive expression of *tbz17* resulted in increased cell size and sucrose production in the leaves [31]. In kiwifruit, AchnbZIP12 was reported to positively regulate the response to ABA-promoted suberization by affecting the expression of *AchnKCS* [32]. In maize, a bZIP transcription factor, OBF1, was revealed to form a complex with UNBRANCHED2 and control inflorescence architecture [33]. To date, the roles of bZIP11 and its homologous proteins in flower senescence are still not well understood.

Petunia is an important ornamental plant with large showy flowers, short growth period, and high genetic diversity [34]. In previous studies, petunia has been adopted as a model plant to elucidate the regulatory mechanisms underlying flower senescence. Some up-regulated transcription factors during the flowering period of petunia were identified through transcriptional analyses and further functionally characterized [13, 35–37]. We have used the tobacco rattle virus (TRV)-based virus-induced gene silencing (VIGS) method with a reporter gene *PhCHS* to characterize the functions of these genes. It resulted in the revealment of a bZIP transcription factor, annotated as *PhOBF1*, which plays a critical role in antiviral RNA silencing [38]. In the course of these studies, we have also found that transcription of *PhOBF1* was significantly induced by several senescence-related hormones, and *PhOBF1*-RNAi and -overexpressing transgenic petunia plants showed variable flower longevity. These findings prompt us to hypothesize that PhOBF1 may function as an important regulator of petal senescence. Here, we elucidate the molecular regulatory mechanism of PhOBF1-mediated flower senescence. Down-regulation of *PhOBF1* accelerated corolla senescence, whereas its overexpression delayed the senescence process. Our data demonstrate that PhOBF1 regulates flower senescence by modulating the GA biosynthesis in petunia.

## Results

### PhOBF1 is phylogenetically related to AtbZIP11 and subcellularly localized in the nucleus

**Figure 1:**
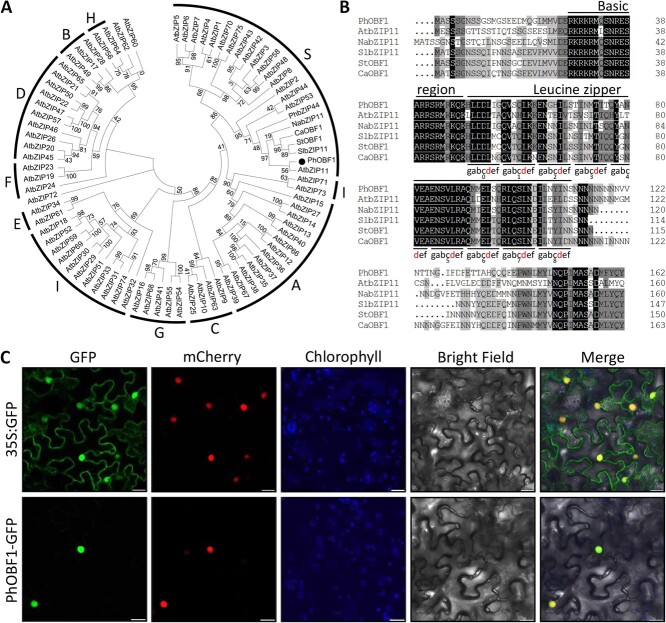
Sequence analysis and subcellular localization of PhOBF1. **A** Phylogenetic tree of PhOBF1 with *Petunia hybrida* PhbZIP44 (Peaxi162Scf00010g00091), *Solanum lycopersicum* SlbZIP11 (XP_004238299), *Solanum tuberosum* StOBF1 (XP_006341974), *Capsicum annuum* CaOBF1 (XP_016568511), *Nicotiana attenuate* NabZIP11 (XP_019245091), and bZIP proteins from *Arabidopsis thaliana*. The sequences of Arabidopsis bZIP proteins were obtained from the Arabidopsis Information Resource. PhOBF1 is highlighted by a solid circle. Bootstrap values are expressed as a percentage of 1000 replicates and shown at branch nodes. **B** Alignment of deduced PhOBF1 amino acid sequence with similar proteins from other plant species. Solid lines indicate the conserved basic region and leucine zipper domains. The heptad repeats of leucine zipper are marked with underlines and gabcdef. The red letters represent the positions of conserved amino acids within the heptad repeats. **C** Transient expression of PhOBF1-GFP fusion protein in *N. benthamiana* leaves. The fluorescent signals were visualized by confocal microscopy 16 h after infiltration. H2B-mCherry was used as a marker to indicate the nuclei. The chlorophyll autofluorescence is displayed in blue color. Scale bars = 20 μm.

To explore the involvement of bZIP genes in flower senescence, one up-regulated transcript encoding a putative ocs element binding factor, namely *PhOBF1* (SGN accession no. Peaxi162Scf00285g00011), was identified during petunia flower senescence through transcriptome sequencing analysis [36]. Phylogenetic analysis revealed a close relationship of PhOBF1 with AtbZIP11 in Arabidopsis, NabZIP11 in tobacco, SlbZIP11 in tomato, StOBF1 in potato, and CaOBF1 in pepper. All these proteins belong to the group S of bZIP transcription factors (Fig. 1A). PhOBF1 and its homologous proteins shared conserved basic region and leucine zipper domains. They displayed a remarkably long leucine zipper domain consisting of up to eight heptad repeats (Fig. 1B) [39]. To analyse the subcellular localization of PhOBF1, the fusion protein PhOBF1-GFP was transiently expressed in tobacco leaves with a nucleus-localized marker H2B-mCherry. Green fluorescent signals of PhOBF1-GFP were observed to overlap with red fluorescent signals of H2B-mCherry (Fig. 1C), suggesting that PhOBF1 was located in the nucleus.

### *PhOBF1* is up-regulated during flower senescence and under different hormone treatments

**Figure 2:**
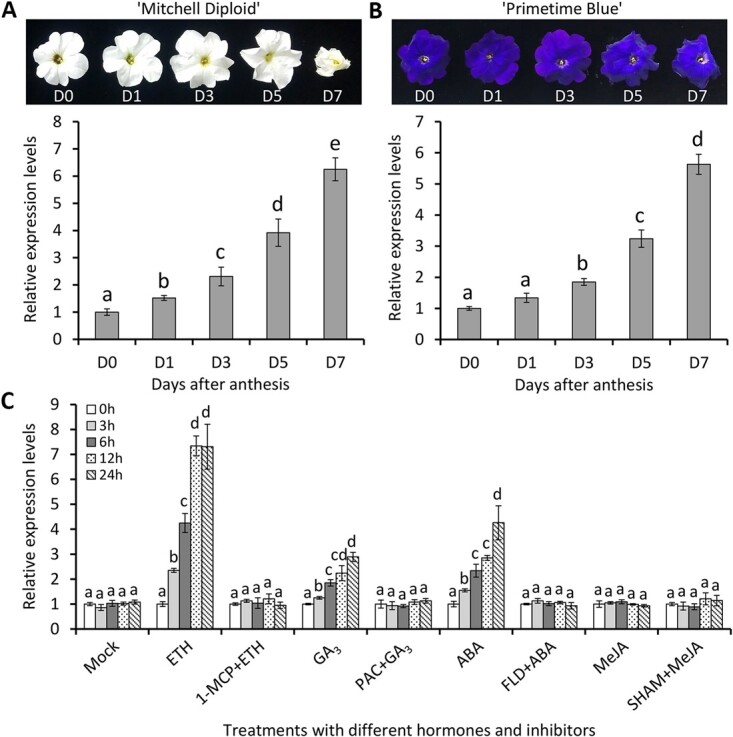
Expression of *PhOBF1* during petunia flower senescence and in response to exogenous hormones. Representative phenotypes and transcript levels of *PhOBF1* in the detached flowers from petunia cultivar ‘Mitchell Diploid’ (**A**) and ‘Primetime Blue’ (**B**) at different opening stages. D0: the day of anthesis, D1, D3, D5, and D7: 1, 3, 5, and 7 days after anthesis. (**C**) Relative expression levels of *PhOBF1* under hormone treatments at intervals. Petunia detached flowers at D0 were treated with 10 μL·L^−1^ gaseous ethylene (ETH), 50 μM GA_3_, 50 μM ABA, and 100 μM MeJA, or with their respective inhibitors 50 nL·L^−1^ 1-MCP, 20 μM PAC, 20 μM FLD, and 40 μM SHAM before hormone treatments. The treatment with water was used as the control (mock). Transcript levels were standardized to *26S rRNA*. Error bars represent standard error of the mean from three biological replicates. Different letters indicate statistical significance as calculated by Duncan’s multiple range test at *P* < 0.05.

Expression profile of *PhOBF1* was examined during flower senescence using quantitative real-time PCR. Transcript levels of *PhOBF1* continued to increase in the detached flowers of both petunia cultivars ‘Mitchell Diploid’ and ‘Primetime Blue’ from anthesis (D0) to 7 days (D7) after anthesis (Fig. 2A and B). As plant hormones are involved in flower senescence, we analysed the transcript profiles of *PhOBF1* in petunia detached corollas treated with various growth regulators. *PhOBF1* were significantly up-regulated following treatments with ethylene, GA_3_, and ABA but not methyl jasmonate (MeJA). The induced transcription of *PhOBF1* by the three hormones was prevented by pre-treatments with their action or biosynthesis inhibitors 1-methylcy-clopropene (1-MCP), paclobutrazol (PAC), and fluridone (FLD), respectively. In addition, the pre-treatment with MeJA biosynthesis inhibitor salicylhydroxamic acid (SHAM) did not affect the transcription of *PhOBF1* (Fig. 2C). Next, we examined the effects of exogenous hormone treatments on flower senescence. Compared to mock control, ethylene and GA_3_ applications accelerated and delayed petal senescence, respectively. The treatment with a combination of the two hormones led to a recovery of ethylene-induced flower senescence (Fig. S1A, see online supplementary material). Specifically, ethylene treatment alone shortened flower longevity by 2.1 days, whereas GA_3_ extended the longevity by 1.9 days. In contrast, the flowers co-treated with ethylene and GA_3_ showed an insignificant variation in flower longevity compared to mock control (Fig. S1B, see online supplementary material), indicating that GAs play an antagonistic role in ethylene-induced flower senescence.

### Silencing and overexpression of *PhOBF1* affect petunia flower senescence

**Figure 3:**
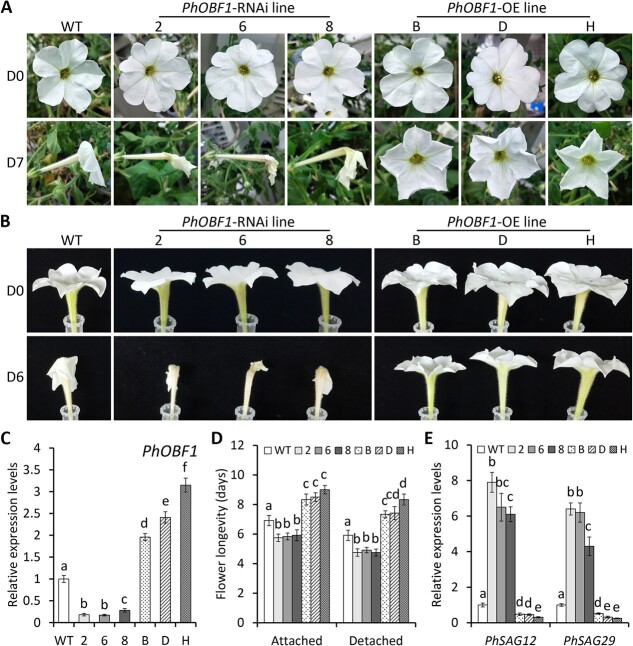
RNAi silencing and overexpression of *PhOBF1* affect petunia flower senescence. **A** Representative phenotypes of attached flowers from wild-type (WT), *PhOBF1*-RNAi, and *PhOBF1*-overexpressing (OE) transgenic petunia lines at anthesis (D0) and 7 days (D7) after anthesis. **B** Representative phenotypes of detached flowers from WT, *PhOBF1*-RNAi, and *PhOBF1*-OE transgenic petunia lines at D0 and D6 after anthesis. **C** Relative expression levels of *PhOBF1* in the attached flowers from WT and *PhOBF1* transgenic petunia plants. The flowers at D0 were collected for quantitative real-time PCR analysis. **D** The longevity of attached and detached flowers from WT and *PhOBF1* transgenic petunia plants. Ten flowers from each of three different plants for each line were counted for longevity evaluation. **E** Relative expression levels of two senescence marker genes *PhSAG12* and *PhSAG29* in the attached flowers from WT and *PhOBF1* transgenic petunia plants. The flowers at D6 after anthesis were harvested for quantitative real-time PCR analysis. *26S rRNA* was used as an internal control. Error bars represent standard error of the mean from three biological replicates. Statistical significance was determined using Duncan’s multiple range test (*P* < 0.05) and shown as different letters.

To investigate the role of *PhOBF1* in flower senescence, we generated transgenic petunia lines with *PhOBF1* RNAi silencing and overexpression using the cultivar ‘Mitchell Diploid’. The flowering phenotypes among different lines were observed at D0 and D7 after anthesis for attached corollas, or at D0 and D6 after anthesis for detached corollas. Compared to wild-type (WT) lines, *PhOBF1*-RNAi lines displayed accelerated flower senescence, while *PhOBF1*-overexpressing lines showed delayed senescence progress (Fig. 3A and B). *PhOBF1*-RNAi and -overexpressing lines displayed a substantial reduction and increase in expression levels of *PhOBF1*, respectively, compared to WT lines (Fig. 3C). Both attached and detached flowers showed similar ranges of flower lifespan variation in transgenic petunia plants. Specifically, silencing of *PhOBF1* shortened flower longevity by 1.0 to 1.2 days. In contrast, overexpression of *PhOBF1* extended flower longevity by 1.4 to 2.2 days (Fig. 3D). Transcript levels of the senescence marker genes *PhSAG12* and *PhSAG29* increased and decreased substantially in *PhOBF1*-silenced and -overexpressing lines, respectively, in comparison to WT controls (Fig. 3E).

### *PhOBF1* is involved in the GA biosynthesis and signaling pathways

**Figure 4:**
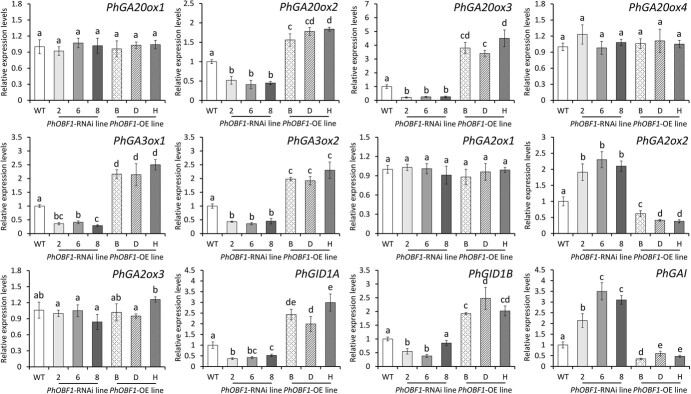
Expression of genes associated with GA biosynthesis and signaling in petunia flowers with *PhOBF1* RNAi silencing and overexpression. Relative expression levels of GA biosynthetic and signaling genes in the attached flowers from wild-type (WT), *PhOBF1*-RNAi, and *PhOBF1*-overexpressing (OE) transgenic petunia lines at 6 days (D6) after anthesis. GA biosynthetic genes include *PhGA20ox1*, *PhGA20ox2*, *PhGA20ox3*, *PhGA20ox4*, *PhGA3ox1*, *PhGA3ox2*, *PhGA2ox1*, *PhGA2ox2*, and *PhGA2ox3*. GA signaling genes include *PhGID1A*, *PhGID1B*, and *PhGAI*. *26S rRNA* served as a reference gene. Error bars represent standard error of the mean from three biological replicates. Significance of difference was calculated using Duncan’s multiple range test (*P* < 0.05) and indicated as different letters.

**Figure 5:**
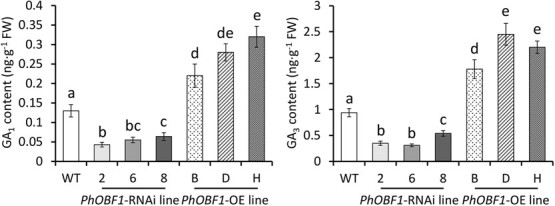
Production of bioactive GAs in petunia flowers with *PhOBF1* RNAi silencing and overexpression. The levels of endogenous bioactive GAs, GA_1_ and GA_3_, in the attached flowers from wild-type (WT), *PhOBF1*-RNAi, and *PhOBF1*-overexpressing (OE) transgenic petunia plants. The flowers at 6 days (D6) after anthesis were collected for GA quantification analysis. Error bars represent standard error of the mean from three biological replicates. Different letters indicate statistical significance as determined by Duncan’s multiple range test at *P* < 0.05.

Genetic transformation of *PhOBF1* in petunia led to significant phenotypic differences in plant height and petal diameter, which were lower in the RNAi lines and higher in the overexpressing lines than in the WT lines (Fig. S2, see online supplementary material). It is commonly known that the plant hormone GAs are responsible for the growth of plant shoots and flowers [37]. To verify the correlation between the phenotypic differences and the GA pathway, we measured expression levels of GA biosynthetic and signaling genes in WT and *PhOBF1* transgenic petunia lines. Three *PhOBF1*-RNAi lines showed significant reduction in transcript abundances of *PhGA20ox2*, *PhGA20ox3*, *PhGA3ox1*, *PhGA3ox2*, *PhGID1A*, and *PhGID1B*, and their transcript levels increased in *PhOBF1*-overexpressing lines. By comparison, *PhGA2ox2* and *PhGAI* were up-regulated in the silenced plants and down-regulated in the overexpressing plants (Fig. 4). Silencing and overexpression of *PhOBF1* did not change the expression of *PhGA20ox1*, *PhGA20ox4*, *PhGA2ox1*, and *PhGA2ox3* (Fig. 4)*.* Consistent with the transcriptional variations, the production of bioactive GAs, GA_1_ and GA_3_, was significantly reduced and increased in petunia flowers with *PhOBF1* RNAi silencing and overexpression, respectively (Fig. 5).

### Exogenous GA treatment inhibits ethylene-mediated petunia flower senescence

**Figure 6:**
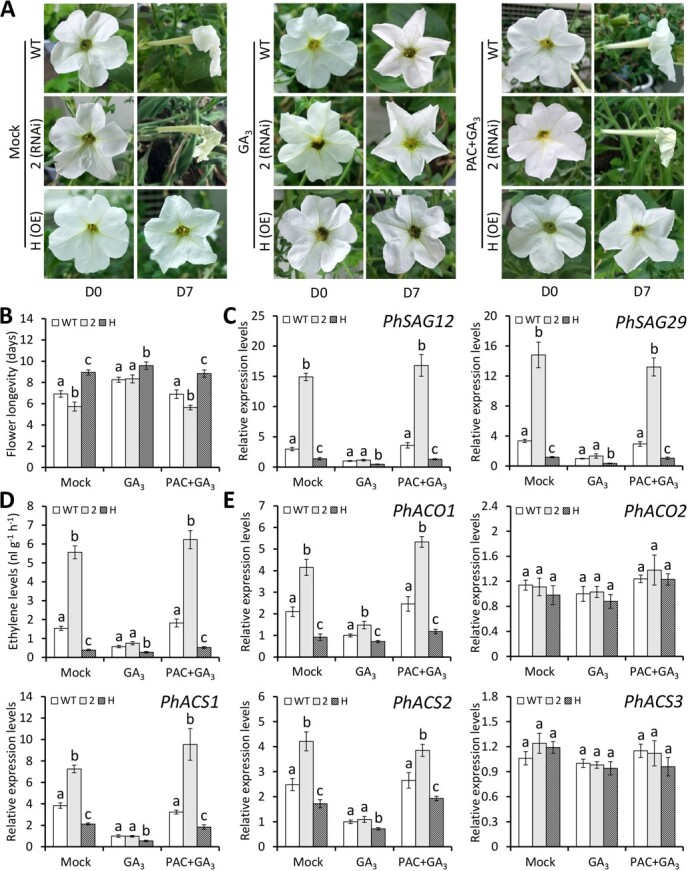
Impact of exogenous GA treatment on flower senescence and ethylene production in *PhOBF1* transgenic petunia plants. **A** Representative phenotypes of attached flowers from wild-type (WT), *PhOBF1*-RNAi, and *PhOBF1*-overexpressing (OE) transgenic petunia lines after treatment with 50 μM GA_3_ or with 20 μM PAC before GA_3_ treatment. The treatment with water was used as the control (mock). The flowers at anthesis (D0) and 7 days (D7) after anthesis were photographed. **B** The longevity of attached flowers from WT and *PhOBF1* transgenic petunia plants after the mock, GA_3_, or PAC/GA_3_ treatment. Ten flowers from each of three different plants for each line were counted for longevity evaluation. **C** Relative expression levels of *PhSAG12* and *PhSAG29* in the attached flowers from WT and *PhOBF1* transgenic petunia plants after the mock, GA_3_, or PAC/GA_3_ treatment. Ethylene production (**D**) and relative expression levels of ethylene biosynthetic genes (**E**) in the attached flowers from WT and *PhOBF1* transgenic petunia plants after hormone treatments. The flowers at D6 after anthesis were harvested for ethylene measurement and gene expression analysis. Transcript abundances were normalized to *26S rRNA*. Error bars represent standard error of the mean from three biological replicates. Different letters indicate statistical significance as calculated by Duncan’s multiple range test at *P* < 0.05.

Given that the transcription of GA biosynthetic and signaling genes and GA content were changed in *PhOBF1* transgenic petunia plants, we speculated that *PhOBF1*-mediated flower senescence may be associated with the GA pathway. We therefore examined the impact of GA_3_ treatment on petal senescence in WT and *PhOBF1* transgenic lines. At D7 after anthesis, we found that the application of GA_3_ alone suppressed the accelerated petal senescence in *PhOBF1*-silenced line (2), and further promoted the delayed senescence in *PhOBF1*-overexpressing line (H). In contrast, the pre-treatment with GA biosynthesis inhibitor PAC did not result in significant petal senescence change in transgenic lines, compared to mock control (Fig. 6A). The mock treatment did not affect flower longevity of WT, *PhOBF1*-silenced and -overexpressing lines. Under GA_3_ treatment, *PhOBF1*-RNAi line had similar flower longevity with WT line, and *PhOBF1*-overexpressing line showed increased flower life (Fig. 6B). In accordance with flower longevity, *PhSAG12* and *PhSAG29* were highly expressed in WT and *PhOBF1*-RNAi lines, and lowly expressed in transgenic line overexpressing *PhOBF1* (Fig. 6C). For the co-treatment with PAC and GA_3_, the overall flower longevity and transcript abundances of two senescence-associated genes in WT and *PhOBF1* transgenic lines were almost identical to those in mock-treated plants (Fig. 6B and C).

To dissect the antagonistic effect between GAs and ethylene, the ethylene release and expression of its biosynthetic genes were examined. Compared to the mock treatment and co-treatment with PAC and GA_3_, the GA_3_ treatment substantially reduced ethylene production in WT and *PhOBF1* transgenic plants. Specifically, similar ethylene levels were detected in WT and *PhOBF1*-RNAi lines after GA_3_ treatment (Fig. 6D), suggesting that GA_3_ application reduced corolla senescence in *PhOBF1*-RNAi line back to the normal levels as in WT line. Correspondingly, the GA_3_ treatment significantly decreased the transcription of several ethylene biosynthetic genes, including *PhACO1*, *PhACS1*, and *PhACS2*, in WT and *PhOBF1* transgenic lines in comparison to the mock treatment and co-treatment with PAC and GA_3_ (Fig. 6E). These findings suggested that PhOBF1 negatively regulates ethylene-mediated corolla senescence by modulating the GA production.

### PhOBF1 directly binds to the promoter of GA biosynthetic gene *PhGA20ox3*

**Figure 7:**
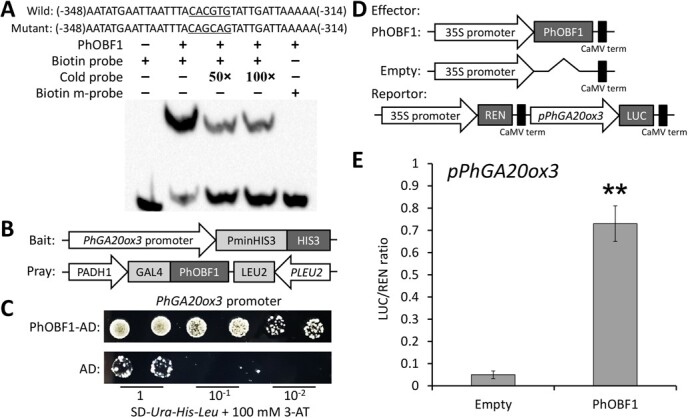
PhOBF1 binds to the promoter of GA biosynthetic gene *PhGA20ox3*. **A** The interaction of PhOBF1 and a biotin-labeled probe through electrophoretic mobility shift assay (EMSA). The probe sequences used for EMSA are shown with the wild-type (wild) *cis*-element and its nucleotide substitutions (mutant) being underlined. Non-labeled probes (cold) at 50- and 100-fold concentrations were used for competition. **B** Schematic diagrams of the bait and pray constructs for yeast one-hybrid assay. **C** Growth of yeast cells transformed with the bait and pray plasmids on the SD-*Ura*-*His*-*Leu* plates supplemented with 100 mM 3-aminotriazole (3-AT) at different dilutions. **D** Schematic diagrams of the effector and reporter constructs for dual luciferase assay. LUC, firefly luciferase; REN, *Renilla* luciferase. **E** Dual luciferase assay of the *PhGA20ox3* promoter (*pPhGA20ox3*). The activation is expressed as a LUC/REN ratio. Error bars represent the standard error of the means from three biological replicates. Statistical significance was determined using Student’s *t* test (*P < 0.05, **P < 0.01) and denoted by asterisks.

To better elucidate the regulatory function of PhOBF1, we searched the promoter sequences of those GA biosynthetic and signaling genes with altered transcription in *PhOBF1* transgenic petunia plants. A G-box motif (CACGTG containing an ACGT core), which is the predicted binding site of PhOBF1’s homolog AtbZIP11 [40], was identified in the *PhGA20ox3* promoter region with 1.5 kb in length (Fig. S3, see online supplementary material). A short DNA fragment bearing the G-box motif in the *PhGA20ox3* promoter served as a probe for electrophoretic mobility shift assay (EMSA). The results revealed a clear binding of PhOBF1 to the biotin-labeled probe, whereas the binding was dramatically suppressed when the unlabeled probe was added (Fig. 7A). To confirm the transactivation of *PhGA20ox3* promoter by PhOBF1, a yeast one-hybrid experiment using the bait and pray constructs was conducted (Fig. 7B). PhOBF1 was found to bind to the *PhGA20ox3* promoter and enhance the growth of yeast cells on the SD-*Ura*-*His*-*Leu* medium supplemented with 3-aminotriazole (3-AT) (Fig. 7C). In addition, we also performed a dual luciferase assay to test the interaction between PhOBF1 and *PhGA20ox3* promoter. A 14.6-fold increase in firefly luciferase (LUC) activity was detected after the co-expression of 35S:PhOBF1 and pPhGA20ox3:LUC (Fig. 7D and E), indicating that PhOBF1 specifically transactivates the *PhGA20ox3* promoter.

### Silencing of *PhGA20ox3* by VIGS accelerates petunia flower senescence

**Figure 8:**
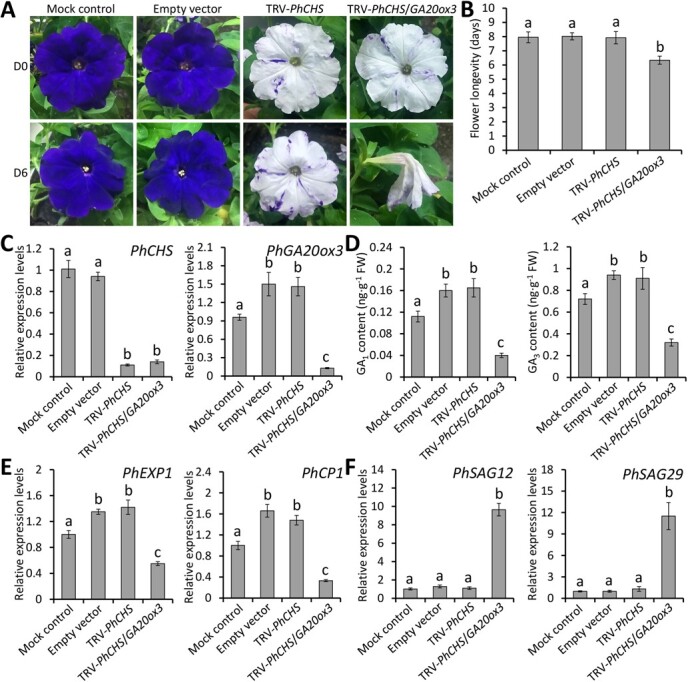
VIGS silencing of *PhGA20ox3* accelerates petunia flower senescence. **A** Representative phenotypes of attached flowers from wild-type (WT) petunia plants infiltrated with non-transformed *Agrobacterium* (mock control), or *Agrobacterium* bearing a TRV empty vector, TRV-*PhCHS*, or TRV-*PhCHS*/*GA20ox3* construct. Photographs were taken at anthesis (D0) and 6 days (D6) after anthesis. **B** The longevity of attached flowers from the mock-, TRV empty vector-, TRV-*PhCHS*-, and TRV-*PhCHS*/*GA20ox3*-infected petunia plants. Ten flowers from each of three different plants for each infection were counted for longevity evaluation. **C** Relative expression levels of *PhCHS* and *PhGA20ox3* in the attached flowers from petunia plants inoculated with mock control and various TRV constructs. **D** Accumulation levels of endogenous bioactive GAs in the mock- and TRV construct-infected attached flowers. Relative expression levels of *PhEXP1*, *PhCP1* (**E**), *PhSAG12*, and *PhSAG29* (**F**) in the attached flowers from petunia plants inoculated with mock control and various TRV constructs. Transcript levels were standardized to *26S rRNA*. Error bars represent standard error of the mean from three biological replicates. Significance of difference was determined using Duncan’s multiple range test (*P* < 0.05) and indicated as different letters.

To further verify the involvement of *PhGA20ox3* in flower senescence, a TRV-based VIGS method was employed to knockdown *PhGA20ox3* in purple-flowered petunia cultivar ‘Primetime Blue’. A 244-bp fragment from the *PhGA20ox3* cDNA was cloned into the TRV vector with a visual reporter gene *PhCHS*. The white-petal phenotypes observed in petunia plants indicated the silencing of *PhCHS*, providing a useful marker for functional analysis of *PhGA20ox3* in floral organs. TRV-*PhCHS*/*GA20ox3*-infected plants showed lower plant height and petal size than the plants infiltrated with the mock, empty vector and TRV-*PhCHS* (Fig. S4, see online supplementary material). Semi-quantitative RT-PCR and quantitative real-time PCR analyses revealed the accumulation of TRV RNA1 and RNA2 in the flowers systemically infected with different TRV constructs (Fig. S5, see online supplementary material). At D6 after anthesis, the plants infected with TRV-*PhCHS*/*GA20ox3* displayed accelerated petal senescence compared to the mock-, empty vector-, and TRV-*PhCHS*-infected plants (Fig. 8A). Silencing of *PhGA20ox3* decreased flower longevity by 1.6 days in comparison to the controls (Fig. 8B). The suppression of *PhCHS* and *PhGA20ox3* transcription in TRV-*PhCHS*/*GA20ox3*-infected flowers was verified by quantitative real-time PCR (Fig. 8C). *PhGA20ox3* silencing resulted in a remarkably reduced production of bioactive GAs, GA_1_ and GA_3_, in TRV-*PhCHS*/*GA20ox3*-infected flowers (Fig. 8D). Two GA-inducible genes *expansin 1* (*PhEXP1*) and *cysteine proteinase 1* (*PhCP1*) were down-regulated in the flowers infected with TRV-*PhCHS*/*GA20ox3* (Fig. 8E). Expression levels of *PhSAG12* and *PhSAG29* in TRV-*PhCHS*/*GA20ox3*-infected corollas were detected to be significantly higher than those in the corollas infected with the mock, empty vector, and TRV-*PhCHS* (Fig. 8F)*.* These observations revealed the critical role of *PhGA20ox3* in PhOBF1-regulated flower senescence.

## Discussion

Flower senescence is closely associated with the economic significance of ornamental plants [41]. The delay of senescence progress is essential for improving the flower quality. It has been indicated that endogenous plant hormones participate in the modulation of flower senescence [42]. In this study, we revealed that a bZIP transcription factor gene, designated *PhOBF1*, plays a crucial role in regulating the senescence process of petunia corollas. PhOBF1 was found to specifically affect the expression of GA biosynthetic gene *PhGA20ox3*, which was functionally characterized as an inhibitor of flower senescence (Figs 7 and 8). Our data suggest a PhOBF1-PhGA20ox3 regulatory module that interferes with the ethylene-mediated senescence process in petunia corollas (Fig. S6, see online supplementary material).

### PhOBF1 functions as a regulator of ethylene-mediated flower senescence in petunia

bZIP family is known to have regulatory functions in many plant-specific processes, such as flower development [43]. In Arabidopsis, the bZIP genes have been reported to control floral inductive signals during the flowering period [44]. AtbZIP14 also known as FD can interact with FLOWERING LOCUS T (FT) to cooperatively regulate flowering time via a direct activation of *APETALA1* (*AP1*) [45]. The *PERIANTHIA* gene encoding a bZIP transcription factor has been demonstrated to be involved in the evolution of flower pattern in the mustard family [46]. A recent report revealed that two bZIP genes, *PaFDL1* and *PaFDL2*, from *Platanus acerifolia* functioned as important regulators of flower initiation and morphology [47]. However, little is known about the roles of bZIP genes in controlling petal senescence. In our transcriptome data, a member of the bZIP family, *PhOBF1*, was significantly up-regulated in senescing flowers, suggesting its potential role in modulating flower senescence. The present results validated the crucial involvement of PhOBF1 in the regulation of flower senescence. Our findings uncovered a novel role of bZIP transcription factor in regulating petunia petal senescence. Aside from *PhOBF1*, transcriptome data showed that the transcription of several other members of the bZIP family was changed during flower senescence of petunia [36]. Further studies should be performed to clarify whether other bZIP members also play regulatory roles in corolla senescence.

Many pieces of evidence have suggested that ethylene is the key hormone controlling the senescence process of ethylene-sensitive flowers [48]. A sharp rise in ethylene production commonly results in the onset of flower senescence [49]. The prominent role of ethylene in controlling petunia corolla senescence has been verified in previous reports [50, 51]. Based on the data presented here, we conclude that PhOBF1 is implicated in the ethylene-mediated flower senescence in petunia. The increased transcription of *PhOBF1* in petunia petals under natural senescence and ethylene treatment supported this assumption (Fig. 2). We noted that GA_3_ treatment also induced the transcription of *PhOBF1* apart from ethylene. This observation seems to contradict the notion that ethylene and GAs promote and delay flower senescence, respectively. One explanation is that PhOBF1 may be implicated in ethylene- and GA-mediated stress responses. It is well known that ethylene and GAs are not only senescence-related hormones but also stress-associated ones [52]. Much evidence has suggested that these two hormones are involved in defense responses to various biotic and abiotic stresses, including virus [53, 54], fungus [55, 56], low temperature [57, 58], and dehydration [59, 60]. We have previously revealed PhOBF1 to be a positive regulator of antiviral RNA silencing. Inoculation with TRV and treatments with cold and drought significantly increased the expression of *PhOBF1* [38]. The potential role of PhOBF1 in defense responses needs to be further examined in the subsequent work. In addition, ethylene and GAs serve as direct hormonal signals for affecting corolla senescence. As a transcription factor, PhOBF1 was presumed to have regulatory function upstream of ethylene and GAs here. However, the impacts of ethylene and GA_3_ treatments on *PhOBF1* expression seem to be a more complex regulatory mechanism, possibly involving hormonal interplay and feedback regulation. Thus, although ethylene and GAs promote and delay flower senescence, respectively, it does not mean that they will have opposite impacts on *PhOBF1* expression. Our results are consistent with a previous report, showing that both treatments with ethylene and CTK, an inhibitor of petal senescence, promoted the transcription of *RhPR10.1* [11].

We also examined the *PhOBF1* expression following the treatment with a combination of ethylene and GA_3_. A slight 1.8-fold increase in transcript abundance of *PhOBF1* was detected at 24 h after the co-treatment compared to mock control (Fig. S7, see online supplementary material). It suggests that the simultaneous application of ethylene and GA_3_ may interfere with their respective inducing effect on *PhOBF1* expression. This result is in accordance with the fact that there is an antagonistic effect between ethylene and GAs [18, 61]. It is highly likely that the antagonism of these two hormones interrupted the functions of ethylene and GAs in the flowers after the combined treatment, thus resulting in an impaired induction of *PhOBF1* expression.

Furthermore, PhOBF1 was identified to be an important regulator of petunia flower senescence through stable genetic transformation (Fig. 3). In most cases, the transcripts with increased expression during flower senescence play positive roles in regulating petal senescence [35]. For example, silencing of *PhFBH4*, one up-regulated gene at different stages of flower senescence, was reported to delay petunia petal senescence, while its overexpression had the opposite effect [37]. However, the contradictory results were shown in this study with reduced and increased expression of *PhOBF1* promoting and suppressing the senescence of petunia petals, respectively (Fig. 3). This finding is consistent with the accelerated senescence in *RhHB6*- and *RhPR10.1*-silenced floral organs in rose [11], and the delayed senescence in *AtERF019*-overexpressing Arabidopsis flowers [62]. In a recent study, a B-box gene *RhBBX28* with increased transcription during rose flower senescence has also been shown to negatively modulate petal senescence [63]. These studies may reveal important mechanisms for inhibiting the senescence process in the flowers.

### PhOBF1 regulates ethylene-induced flower senescence by modulating GA content

The changed accumulation of bioactive GAs (GA_1_ and GA_3_) and transcription of GA biosynthetic and signaling genes were observed in *PhOBF1* transgenic petunia plants (Figs 4 and 5), implying that *PhOBF1*-mediated flower senescence may be dependent on the GA pathway. A number of reports have suggested the roles of bZIP transcription factors in regulating the responses to GA signals. For instance, the tobacco RSG protein with a bZIP domain has been demonstrated to modulate the GA homeostasis via the feedback regulation of *NtGA20ox1*, a gene responsible for synthesizing bioactive GAs [64]. The latest finding showed that the translocation of SWIZ, a bZIP protein from *Brachypodium distachyon*, into the nucleus was negatively affected by GA biosynthesis, and its overexpression has been found to increase transcript abundance of *GA20ox4* [65]. In the present study, we revealed that PhOBF1 regulated the GA production by specifically activating the downstream gene *PhGA20ox3*, whose silencing resulted in shortened flower longevity (Figs 7 and 8). Our data supported the hypothesis that PhOBF1 participates in ethylene-induced floral senescence through the modulation of GA pathway. Application of GA_3_ was observed to block the ethylene function in petal senescence by reducing the transcription of a few ethylene biosynthetic genes: *PhACO1*, *PhACS1*, and *PhACS2* (Fig. 6D and E). It is in accordance with the earlier research that the role of GAs in corolla senescence was antagonistic to that of ethylene [18, 66]. Besides, we found that the combined treatment with ethylene and GA_3_ resulted in an insignificant change in flower longevity compared to mock control (Fig. S1, see online supplementary material). It demonstrated that GA_3_ application inhibited ethylene-induced petal senescence, further validating the antagonism between ethylene and GAs. However, GAs and ethylene have been indicated to co-regulate the epidermal cell death of rice [67] and bud viability of larch [68] in a synergistic manner, suggesting that the interaction between these two hormones may vary among different plant organs or tissues.

It has been reported that Arabidopsis plants constitutively expressing *AtbZIP11*, a homolog of *PhOBF1*, showed inhibited plant growth compared to WT plants [28, 40]. However, we found that *PhOBF1* overexpression resulted in increased plant height and petal size (Fig. S2, see online supplementary material). We re-checked the sequence alignment of PhOBF1 and AtbZIP11 (Fig. 1B) and observed that the similarity between them is only 47.5%. In comparison with AtbZIP11, PhOBF1 displayed a large number of amino acid variations even in its conserved domain, suggesting a high sequence polymorphism between the two proteins. Many pieces of evidence have revealed that amino acid variations commonly result in functional divergence among different proteins in plants [69, 70]. This may explain the phenotypic differences between *PhOBF1*- and *AtbZIP11*-overexpressing transgenic plants. In addition, AtbZIP11 or its homolog has been shown to function as an important regulator of sugar and proline metabolism in plants [28, 31, 40]. It has been uncovered that sugar contributes to the prolongation of flower life due to the suppression of ethylene biosynthesis or sensitivity to ethylene [71]. Proline may play a crucial role in the regulation of petal senescence by affecting energy depletion and reactive oxygen species accumulation [72]. Thus, whether PhOBF1 is involved in the regulation of flower senescence by modulating sugar and proline metabolism requires further exploration.

The bZIP transcription factors are known to activate or inhibit the expression of genes whose promoter sequences contain a consensus ACGT *cis*-acting element [73]. Consistent with this notion, PhOBF1 was found to directly bind to the G-box motif (CACGTG) containing a core ACGT sequence (Fig. 7A). In Arabidopsis, it has been shown that some bZIP proteins, such as AtABI5 [74], AtbZIP11 [40], AtbZIP53 [75], AtbZIP67 [76], AtHY5 [77], and GBFs [78] also target the G-box motif. Other members of bZIP family exhibit variable DNA binding sites with different nucleotides flanking the core ACGT element. For example, the ABRE-binding factors belonging to one group of bZIP transcription factors can specifically bind to the PyACGTGG/TC motif [79]. AtbZIP10 was reported to shuttle between the nucleus and the cytoplasm for binding to G- and C-box (GACGTC) motifs, respectively [80]. ThbZIP1 from *Tamarix hispida* was also found to have multiple binding properties to the C-, G-, and A-box (TACGTA) sequences [81]. Indeed, we identified a number of core ACGT elements in the *PhGA20ox3* promoter region besides the G-box motif. The possibility of binding to those additional ACGT elements by PhOBF1 requires further examination in the future.

### PhOBF1 may be involved in the complex hormonal crosstalk

Apart from ethylene and GA_3_, exogenous treatment with ABA increased expression levels of *PhOBF1* in petunia petals (Fig. 2C). ABA is generally considered as a promoter of senescence in many ethylene-sensitive flowers such as rose [14] and petunia [13]. The interplay between ABA and ethylene has been suggested in some previous reports. ABA treatment inhibited ethylene production in hibiscus flowers [23], while the ABA-induced flower senescence in carnation was attributed to the activation of ethylene synthesis [82]. However, a contrasting report showed that the ABA treatment increased flower sensitivity to ethylene and transcription of ethylene pathway-related genes [83]. In petunia, ABA seems to be a downstream product of the ethylene pathway during flower senescence, because the interruption of ethylene signaling was shown to block ABA accumulation in senescing petals [84]. In addition, an antagonistic effect of GAs on ABA existed in rose flowers, with the reduced GA accumulation accelerating ABA-promoted petal senescence [18]. Based on the induced expression of *PhOBF1* by ABA, we suppose that ABA may play a role in PhOBF1-regulated petunia corolla senescence. It remains to be investigated whether PhOBF1 participates in the ABA-stimulated senescence process, and the crosstalk among ABA, ethylene, and GAs during flower senescence of petunia is still unknown.

It is worth mentioning that endogenous hormones have proven to be critical factors in regulating plant responses to biotic and abiotic stresses. Given the impacts of several stress-associated hormones including ethylene, GA_3_, and ABA on *PhOBF1* expression, we hypothesized that PhOBF1 may be essential for the defense against various environmental stimuli. In support of this hypothesis, we have previously reported an essential role of PhOBF1 in RNA silencing-mediated antiviral defense possibly by activating the salicylic acid biosynthesis pathway [38]. We also found that silencing and overexpression of *PhOBF1* reduced and increased, respectively, VIGS silencing efficiency and resistance to TRV and tobacco mosaic virus infections. In particular, the down-regulation of *PhOBF1* by TRV-based VIGS resulted in a substantially impaired development of *PhCHS*-silenced white-petal phenotype [38]. That is the reason why we did not use the TRV-*PhCHS* system to characterize the function of PhOBF1 in petunia corolla senescence. Moreover, the treatments with cold and dehydration dramatically increased the transcription of *PhOBF1* [38]. Interestingly, AtbZIP11 from Arabidopsis has been revealed to negatively modulate the resistance to *Pseudomonas syringae* infection [85]. It appears likely that the involvement of PhOBF1 in plant defense responses may be a complicated process.

In conclusion, our findings demonstrate that PhOBF1 plays a crucial role in flower senescence by modulating the GA biosynthesis. *In vitro* and *in vivo* promoter-binding tests confirmed the specific regulation of *PhGA20ox3* by PhOBF1. The identification of *PhOBF1*, a differentially-expressed gene from transcriptome data, provides a valuable genetic solution to extend flower longevity of petunia through genetic engineering. An integrative method of high-throughput transcriptome sequencing and VIGS system is needed to find more promising targets for controlling the senescence of petunia flowers. To further dissect the regulatory network of PhOBF1 in flower senescence, an extensive multi-omics data analysis using *PhOBF1* transgenic petunia materials should be performed in future work.

## Materials and methods

### Plant materials and growth conditions

Two petunia (*Petunia* × *hybrida*) cultivars, ‘Mitchell Diploid’ and ‘Primetime Blue’ were used as experimental materials. Their seeds were obtained from Goldsmith Seeds Inc. (Santa Clara, CA, USA). Of them, ‘Mitchell Diploid’ was used for stable genetic transformation assay, while ‘Primetime Blue’ was used for TRV-based VIGS assay. Petunia seeds were sown in a tray containing the soil mix with a ratio of peatmoss to perlite being 2:1. After germination, they were placed into an artificial climate chamber under conditions of 16 h light/8 h dark and 25°C day/20°C night. Four-leaf-stage plantlets were transferred to small plastic pots for a continuous cultivation. Upper leaves from petunia plants were used as the explants for *Agrobacterium*-mediated stable genetic transformation. The plantlets at the six-leaf stage were used for VIGS inoculation with mock control and various TRV constructs. Flower longevity of each plant was determined as the duration from full opening of the petal to its complete wilting [13]. The flowers at D0 were used for various hormone treatments. The corollas at various stages were sampled to determine the levels of gene expression and endogenous hormones.

### Identification of *PhOBF1*

A cDNA fragment of *PhOBF1* containing the complete coding region was identified among up-regulated genes from transcriptome analysis of petunia flower senescence [36]. Its nucleotides were converted into amino acids using the translation tool on the ExPASy web server (http://web.expasy.org/translate/). Amino acids of PhOBF1 were used for searching against Sol Genomics Network (https://solgenomics.net/organism/Petunia_axillaris/genome) and NCBI GenBank (http://www.ncbi.nlm.nih.gov/structure/cdd/wrpsb.cgi) databases to identify its homologous proteins from petunia and other plant species, respectively. The conserved bZIP domains within the amino acids of PhOBF1 and its homologs were identified through the Conserved Domain Search Service on the NCBI web server. Comparison of multiple protein sequences was conducted using the DNAMAN alignment tool (version 8.5). Phylogenetic relationship was analysed through the MEGA tool (version 6.0.6).

### Subcellular localization

The full-length coding region of *PhOBF1*, whose termination codon was removed, was amplified from WT petunia cDNA. It was then introduced into the binary pCAMBIA1301-GFP vector between *Bam*HI and *Sal*I restriction sites. The 35S:PhOBF1-GFP fusion construct under the control of CaMV 35S promoter was generated. The pCAMBIA2300-35S-H2B-mCherry vector with the red fluorescent marker mCherry fused to histone 2B was used as a reference for nuclear localization. 35S:PhOBF1-GFP/35S:H2B-mCherry or 35S:GFP/35S:H2B-mCherry were co-transformed into tobacco leaves using a biolistic PDS-1000He delivery system (Bio-Rad, Hercules, CA, USA). The plants were incubated at 25°C for 16 h in the dark. Subsequently, the transformed cells were visualized for fluorescent signals under a TCS SP8 confocal microscope (Leica, Solms, Germany).

### Treatments with various hormones

The whole flowers with short pedicels were cut from the petunia plants at D0. They were placed immediately into small vials with distilled water or hormone solutions. For ethylene treatment, 10 μL·L^−1^ gaseous ethylene was insufflated into an airtight transparent box containing the flowers. For 1-MCP and ethylene treatments, the corollas were initially exposed to 50 nL·L^−1^ 1-MCP for 3 h prior to the application of ethylene. For other non-gaseous hormones, the flowers were inserted into the vials containing 50 μM GA_3_, 50 μM ABA, and 100 μM MeJA. For the combined treatments with their respective inhibitors, 20 μM PAC, 20 μM FLD, and 40 μM SHAM were firstly applied to the flowers for 3 h, respectively. The corollas were harvested at intervals (0, 3, 6, 12, and 24 h) post treatment. For the combined treatment with ethylene and GA_3_, the flowers inserted into the vials containing 50 μM GA_3_ were simultaneously exposed to 10 μL·L^−1^ gaseous ethylene. To examine the effects of hormone treatments on petal senescence, the flowers were treated with ethylene, GA_3_, and a combination of the two hormones as mentioned above, and photographed at 0 and 6 days after treatments. To check the impact of GA treatment on PhOBF1-mediated flower senescence, the corollas from WT and *PhOBF1* transgenic lines at D0 were sprayed with 50 μM GA_3_ or 20 μM PAC before GA_3_ application. The flowers were harvested at D6 after anthesis. The treatment with deionized water was used as mock control.

### Generation of transgenic petunia plants

The complete open reading frame (ORF) region of *PhOBF1* was amplified and ligated into the pGSA1403 vector to generate the overexpression construct, while a 329-bp fragment in both forward and reverse orientations was introduced into the pGSA1285 vector to form the RNAi construct as previously described [38]. Electroporation was used to transform the recombinant plasmids into *Agrobacterium tumefaciens* strain LBA4404 using a Gene Pulser apparatus (Bio-Rad, Richmond, CA, USA) at 2.5 kV and 400 Ω. *Agrobacterium*-mediated leaf-disk method was used for stable genetic transformation according to a previously described protocol [86]. The positive transgenic plants with kanamycin resistance were screened on the MS plates. PCR amplification was performed to confirm the integration of *PhOBF1* cDNA fragment into the petunia genome. Expression levels of *PhOBF1* in the flowers of WT and transgenic petunia plants at D0 were examined by quantitative real-time PCR. Three representative lines based on transcript levels of *PhOBF1* in the RNAi or overexpression assay were selected for further experiments.

### Semi-quantitative RT-PCR and quantitative real-time PCR

Total RNA isolation was conducted on petunia flowers through a TRIzol-based method. For RNA purification, RNase-free DNase I (Promega, Madison, WI, USA) was added to eliminate contaminating DNA at 37°C for 30 min. About 2–5 μg of total RNA were used for reverse transcription to synthesize cDNA samples using a PrimeScript RT reagent kit (Takara, Otsu, Shiga, Japan). Semi-quantitative RT-PCR was performed with Taq DNA polymerase (TaKaRa, Otsu, Shiga, Japan) in 25 μL volumes following the manufacturer’s instructions. The resulting products were analysed by 1.5% agarose gel electrophoresis using GelRed as a nucleic acid staining dye. The gene bands were visualized and photographed under ultraviolet light with a Gel Doc XR+ imaging system (Bio-Rad, Hercules, CA, USA). Abundance of mRNA was detected by quantitative real-time PCR. The reactions were run in a LightCycler instrument (Roche Diagnostic, Basel, Switzerland) using the SYBR Green Master Mix (Applied Biosystems, Foster City, CA, USA). Transcript levels of genes were standardized to the constitutively expressed *26S rRNA*. Analysis of relative expression data was performed based on the comparison of threshold cycles in PCR reactions [87]. Three independent RNA samples were used for expression analysis.

### Measurement of gibberellins and ethylene

GA levels were measured according to a previously described protocol [88]. In brief, the floral samples were frozen and powdered using a milling tool in the presence of liquid nitrogen. Extraction was conducted using 80% methanol with internal standards GA_1_ and GA_3_ at 4°C for 30 min. The aqueous phase was collected and further extracted with the solution containing EtOAc (pH = 3.0) and K-Pi buffer (pH = 8.5). After purification by chromatography on Sep-Pak C18 column, the eluant was evaporated to dryness with nitrogen at 40°C and re-dissolved in 80% methanol. After purification through a 0.45 μm filter, the solution was detected by HPLC electrospray ionization tandem mass spectrometry (HPLC-ESI-MS/MS). The production of gaseous ethylene was measured as previously described [13]. The flowers with the pedicels were harvested and inserted into the vials with sterile water. They were placed into a 250-mL container and maintained at 25°C for 3 h. A 2-mL hypodermic syringe was used for injecting the samples into a gas chromatographic apparatus (Agilent, Palo Alto, CA, USA) for quantification of ethylene release. Three biological replicates were used with at least five flowers in each replicate.

### Electrophoretic mobility shift assay

EMSA experiment was conducted using a previously reported method [89]. The ORF sequence of *PhOBF1* was introduced into the pET28a vector to form the pET28a-PhOBF1 plasmid, which was then transformed into *Escherichia coli* Rosetta (DE3) cells. Next, 0.1 mM isopropylthio-β-galactoside was added to induce the expression of His-tagged PhOBF1 protein. An ultrasonic device was applied to disrupt the bacterial cells for protein release. The protein samples were extracted and purified by passing through the HisTrap HP column. A 35-bp biotin-labeled DNA fragment in the promoter of *PhGA20ox3* was synthesized and annealed, which was used as WT or mutant probe. The WT probe with no labeling was referred to as the binding competitor (Table S1, see online supplementary material). A LightShift EMSA Optimization and Control Kit (Pierce, Thermo Fisher Scientific, MA, USA) was used for the interaction of protein and DNA. The protein-DNA complex was separated by 6% non-denaturing polyacrylamide gel electrophoresis, and then transferred to a 0.45 μm nylon membrane using an electrophoretic transfer apparatus (Bio-Rad, Hercules, CA, USA). After conjugation and purification, the binding signals on the membrane were visualized and photographed in the imaging system as mentioned above.

### Yeast one-hybrid assay

A 493-bp DNA fragment harboring PhOBF1-bound *cis*-acting element in the *PhGA20ox3* promoter was amplified using a primer pair (Table S1, see online supplementary material). The product was cloned into the pHIS2 vector for the bait construction. The coding sequence of *PhOBF1* with full length was introduced into the pGADT7-Rec vector harboring the GAL4 activation domain, which was regarded as the pray construct. The recombinant plasmids were co-transformed into *Saccharomyces cerevisiae* strain Y187. The positive colonies were selected and identified for liquid culture, which was diluted with LB media to 10 and 100 times. For each dilution, a small amount of cells were spotted on the SD-*Ura-His-Leu* plates by addition of 100 mM 3-AT. The activation of *PhGA20ox3* promoter by PhOBF1 was verified based on the proliferation rates of yeast cells.

### Dual luciferase assay

The assay was carried out according to a previously described method [90]. The complete coding sequence of *PhOBF1* and 1500-bp sequence of *PhGA20ox3* promoter were jointed with the pGreenII62-SK and pGreenII0800-LUC vectors, respectively. For the effector, the CaMV 35S promoter was used to drive the expression of PhOBF1. For the reporter, *PhGA20ox3* promoter and 35S promoter were employed to activate the transcription of LUC and *Renilla* luciferase (REN), respectively. The specific primers used are listed in Table S1 (see online supplementary material). The cells of *A. tumefaciens* strain GV3101 containing the constructed plasmids were used to co-inoculate young petunia leaves. The enzymatic activities of both LUC and REN were examined through a luminometer (Männedorf, Switzerland), and expressed as the LUC/REN ratio.

### Virus-induced gene silencing

The TRV-*PhCHS* plasmid containing a 194-bp fragment of *PhCHS* was constructed as previously described [91]. To obtain the TRV-*PhCHS*/*GA20ox3* construct, a *PhGA20ox3* fragment with 244 bp in length was inserted into the *Sac*I–*Xho*I sites of TRV-*PhCHS* vector. The plasmids were electro-transformed into *A. tumefaciens* strain GV3101. *Agrobacterium* cells with kanamycin resistance were cultured in 15-mL LB media by addition of 40 mg·L^−1^ kanamycin and other reagents at 28°C for 48 h. The cultures were centrifuged at 4000 rpm for 15 min to harvest the cells. The reagents containing 200 μM acetosyringone, 10 mM MES, and 10 mM MgCl_2_ were used to resuspend the harvested cells, whose concentration was then adjusted to an OD_600_ of 4.0. After 3 h of gentle shaking at room temperature, the mixture of TRV1- and TRV2-transformed bacteria was used to inoculate the leaves of petunia plantlets as previously described [38]. Three biological replicates were used with five plantlets in each replicate.

## Supplementary Material

Web_Material_uhad022

## Data Availability

All data supporting the conclusions of this work are present in the paper or its Supplementary material files.
