# Supplementary material for: A petunia transcription factor, PhOBF1, regulates flower senescence by modulating gibberellin biosynthesis
Source: Hortic Res. 2023 Feb 16;10(4):uhad022. doi: 10.1093/hr/uhad022 (PMC10541524; doi:10.1093/hr/uhad022)
Supplement: Web_Material_uhad022 [file web_material_uhad022.docx]

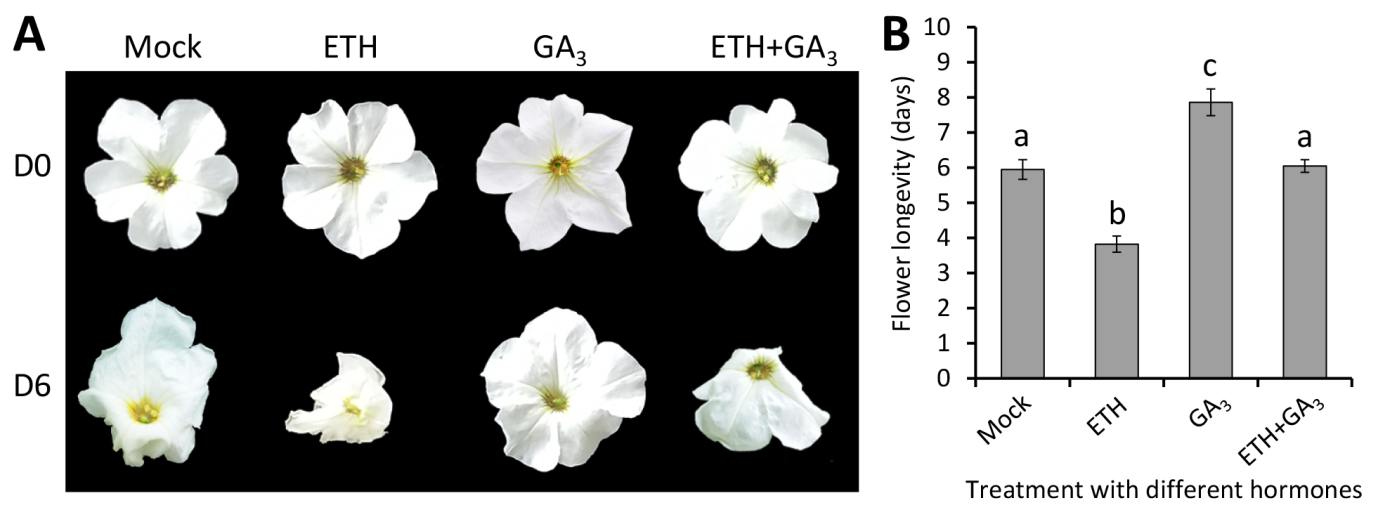


**Figure S1. Effects of exogenous hormone treatments on the senescence of petunia flowers.** **(A)** Representative phenotypes of detached flowers treated with 10 μL·L^–1^ ethylene (ETH), 50 μM GA_3_, and a combination of ETH and GA_3_. The flowers at anthesis (D0) were used for the treatments. Photographs were taken at 0 and 6 days after treatments. **(B)** The longevity of detached flowers treated with various hormones. Ten flowers for each treatment were counted. The treatment with water was used as the control (mock). Error bars represent standard error of the mean from three biological replicates. Different letters suggest statistical significance as determined by Duncan’s multiple range test at *P* < 0.05.


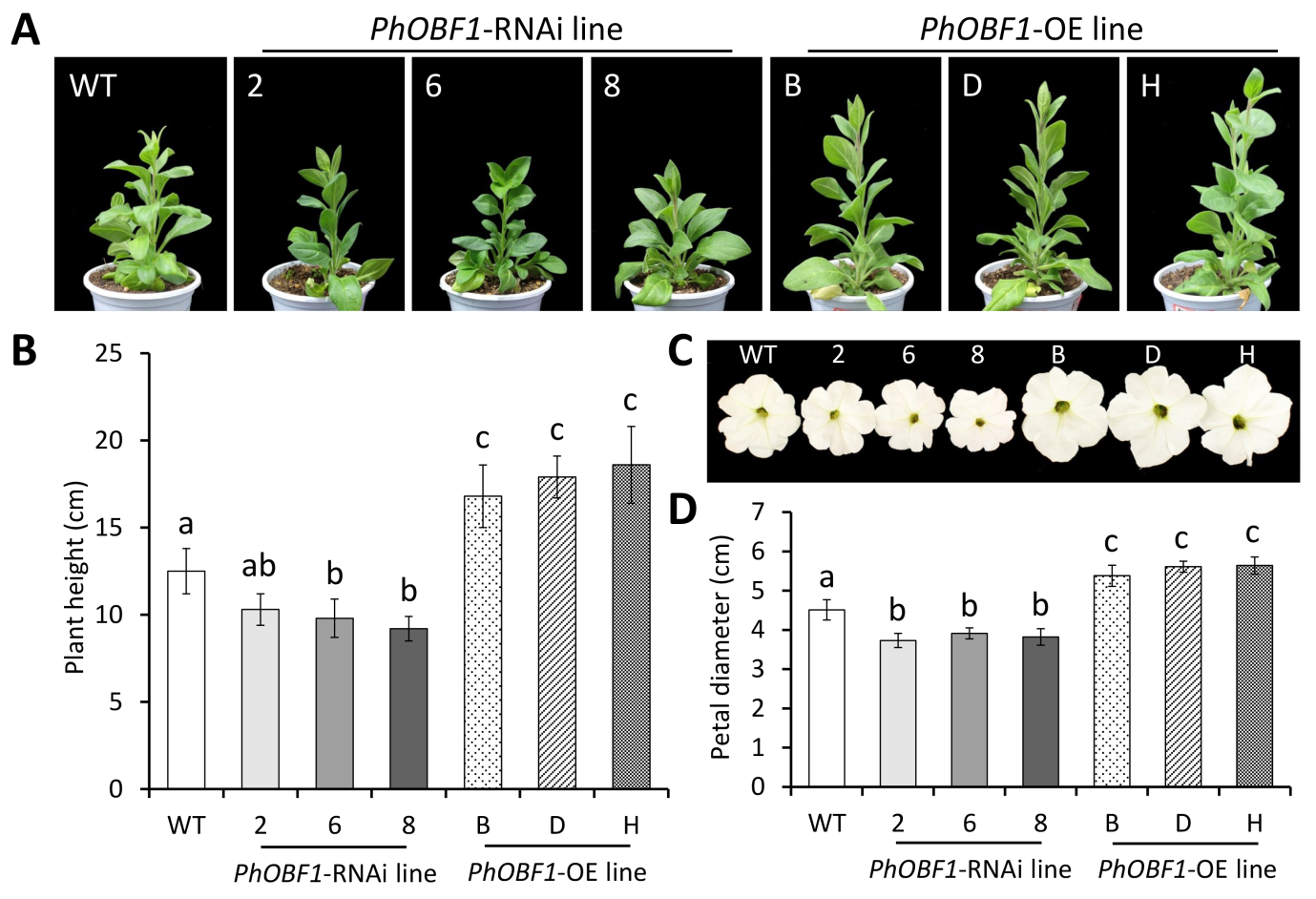


**Figure S2. Phenotypic traits of transgenic petunia plants with *PhOBF1* RNAi silencing and overexpression.** **(A)** Representative phenotypes of wild-type (WT), *PhOBF1*-RNAi, and *PhOBF1*-overexpressing (OE) transgenic petunia plants. The plants at 60 days after germination were photographed. **(B)** Plant heights of WT and *PhOBF1* transgenic petunia plants at 60 days after germination. Representative phenotypes **(C)** and petal diameters **(D)** of the flowers from WT and *PhOBF1* transgenic petunia plants at anthesis (D0). Error bars represent standard error of the mean from three biological replicates. Different letters indicate statistical significance as calculated by Duncan’s multiple range test at *P* < 0.05.

GCTTGGTATTTGAGACTAAAGATAAAGTTGTTTCATAGGATGATTGATATCCTATCATATTCCTCAAACATTTCAATCTATATTTGAGATAAACACGTAAGTGGTTTACTTAGCAATGTTTACTTATTTGAATACATAGTTGTTTACTTTTCTTTAAACTCATTATTTTATAGTTATATCTCTTTTCGTGCAATTTGCTTTAGAGAGAGAGAGAGAGAGTGTGTGTAAAAGACTACTTATATATTAACCAATGAGAAAGAAAATGAATACATATCAGTACATATGAAACCGTGATATGCTTTATCAATAGACGGTGATTTTACAATCCTTTCTAGATAATTGTTAGTTTAAAATAAGCATGATTAGATAAAATCTTTATGCTTATCGTTTTAGGTGAGTGAGATTTCATGTCAAATCGTGAGAATGCCAACATCTGTATTGGTAATTCATATGAAATGGTTAACAAAATATTCATAACTTGGGGTGTCAAAAGGTATACGAACTGCAAAAATTGATGAACATTGGCTGTTAAGTAGAATTTTTTCAGCTTATGGTAAATTTCTAAATGAGAAACACTATGAGGGGTAGACTTGAATATTTGAATTAAGTGTGATAAGTTTTGAAATATAAATTGAAAATTAATATGTTTGAATTGGAGATACGAAGATAAGTCAAAATAAAAAACAAATGTGAAATTGAGTTCTTAATGATAATATAATGCACATTATTGTGTTGATAGCTGTTTTCCTACTCTAAAGTCTACACTAGAATGGCAATGAAAAACATTTGAGAAAGGCTTCAGTTATAAGAATTGAAAAATCAATAGATAACATAAGTATCAATACTTTGTTGTAAACCTTGCATGCTATGTTTAAAAGGATTTGGAATTTCAAGAAAGAGTATATTGTATACACCAAGTTTTACACTTTATTAGAACGGCTATAAGAATTAGATTTAGTGTGCAGTTAAATAAGATAATAATTTGCATGATCGGTTTTCTTTGTATATAGTTTAATTGACTATTTTAAATTATTGGAGTTAAGCTTAGTAACAACTTCTATTAATCTATTCTATAATCTAAAAAGAAAATTTATTTTTAAATTCTATTGTGCCTTTCAGAGCTATTTGACTCTATTTATTTCTTTGAACGCGTAAAATTCTAATTTTAAATACATTTGAGAGACACAAAATGATGGTTCAAATTTAATTTGAAAAAAACTAATAGTTCATATCGAATTTGTCGAAATTTCAACATGTAATGTGCTCAAATAAAAGAATATATTTTAGTGATTAATTTCATTTATTATTTGACCGCGTATTGCGTGGGTGCTATACTAATTAGGATAATATTAATTTTTCCCGCTTAATTAATTTTTGCATTAATATTTATTCAACTGCAGAAATTCAAAGGAACATATTCATTAATCTGTTCTTTAGTCATCAGTCAAAACTCCAAGCTAACACTGGCTCCATCACAAGTCTATGGATTGAAGTCTTTTCCTATACCCCATGTGAATGTTGGATACTTCATGTTTTTGGGCTGCCATTTTTATAGTTTTAGGATCATTAATTATATGCTCAATTACGTCTTTTTTTTTTCTTTTCAGTTTCATTCTTTTTGTCGAGATCTCGTGGGAGTGTTTTTCCCCTTAAATATGAATTAATTTACACGTGTATTGATTAAAAAACGTTTTCCAAAGAAAAAATCGTCATATTTAATTTTAGAGGTTGTCTTAGTTACGAAGAATCCTAGAGATTCCTCAATTCGGACGTACATTGGTGGCAGCAACGACAACTCAAATAGGATTCACCAAAATAGCTAGGTCTTGACACACAATGCAAGATTCCATTTTTCTTTTTCTAATCTCTCAATTATCTCAAGCAAGAAAAGGACACATCATCAAATTTCTGTATATATAATACTATAATTGATCCAAAAGAATTTTATGTAGAGAGACACAAATCACGTTAATCAGTCACAGCAATATTATTTTCTTACA***ATG***GCACCAGCTGGAATAGAAGAAGAGCAAATTGACACACTTTCAATGAATAATGGCAACAAAATGCAAG

**Figure S3.** **The promoter sequence upstream of petunia *PhGA20ox3* coding region.** The putative binding motif of PhOBF1 is marked in square. The other ACGT elements are underlined. Bold italic types indicate the start codon of translation. The probe used for electrophoretic mobility shift assay is shaded in grey.


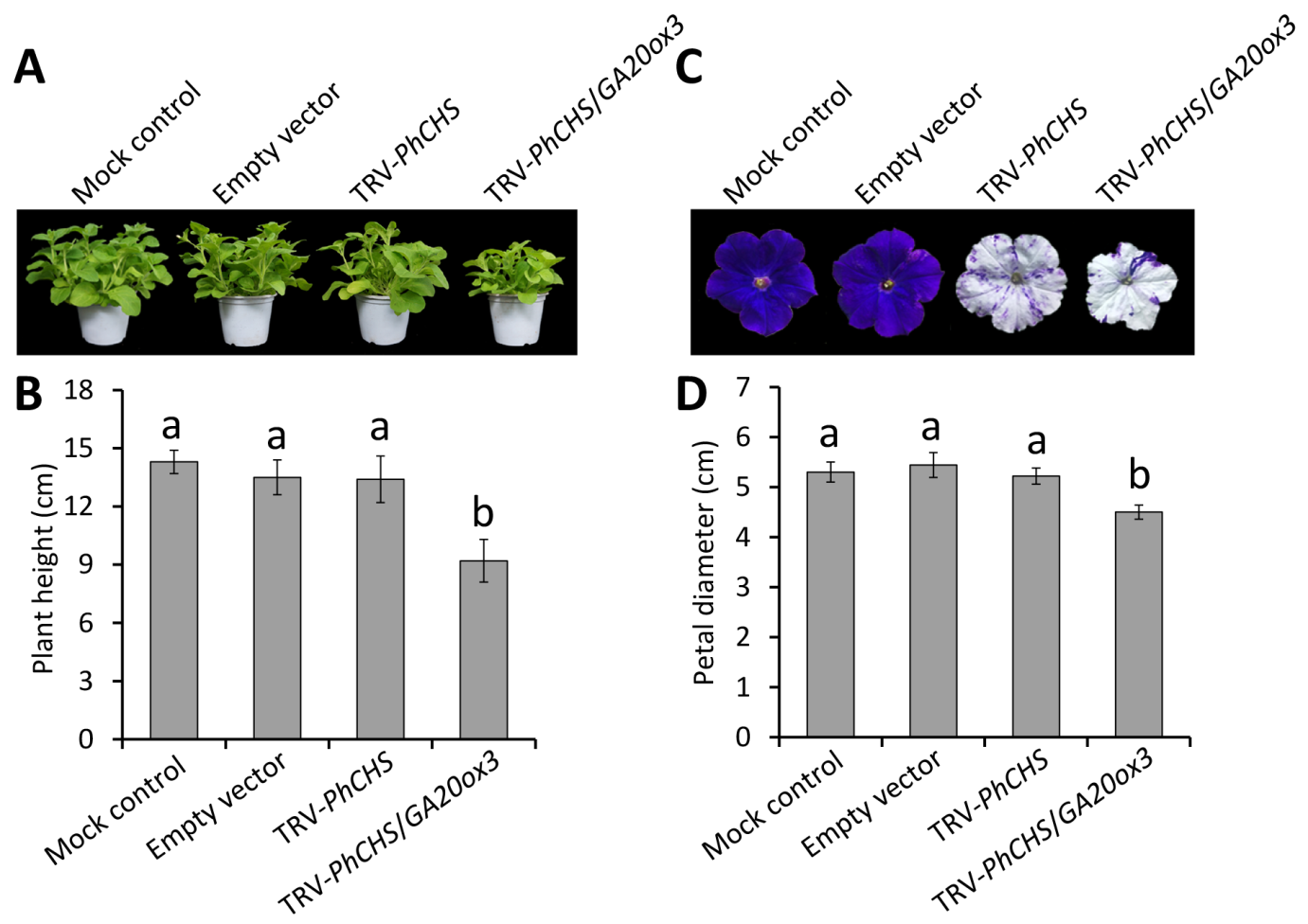


**Figure S4. Phenotypic traits of petunia plants with VIGS silencing of *PhOBF1*. (A)** Representative phenotypes of wild-type (WT) petunia plants infiltrated with non-transformed *Agrobacterium* (mock control), or *Agrobacterium* bearing a TRV empty vector, TRV-*PhCHS*, or TRV-*PhCHS*/*GA20ox3* construct. The plants at 50 days after germination were photographed. **(B)** Plant heights of WT petunia plants infiltrated with mock control and various TRV constructs at 50 days after germination. Representative phenotypes **(C)** and petal diameters **(D)** of the flowers from WT petunia plants infiltrated with mock control and various TRV constructs at anthesis (D0). Error bars represent standard error of the mean from three biological replicates. Different letters suggest statistical significance as determined by Duncan’s multiple range test at *P* < 0.05.


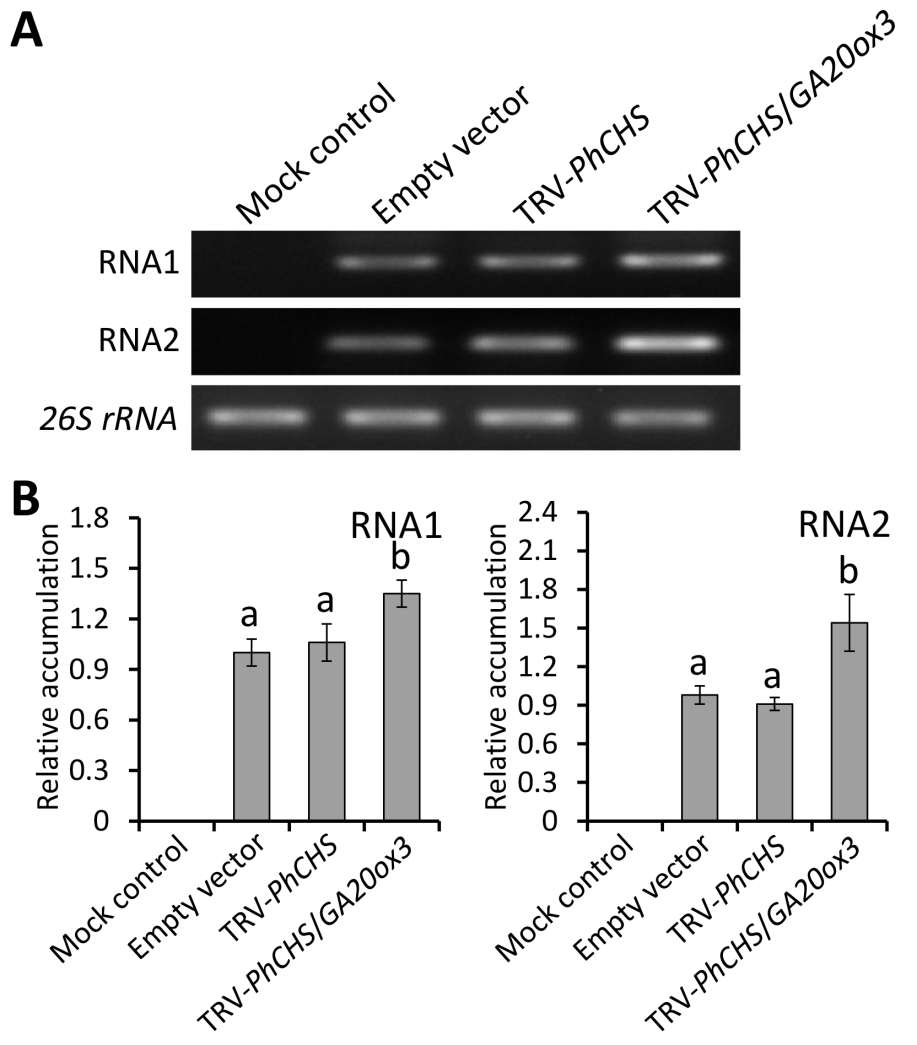


**Figure S5. TRV accumulation levels in the flowers systemically infected with different TRV constructs.** Semi-quantitative RT-PCR **(A)** and quantitative real-time PCR **(B)** analyses of TRV RNA1 and RNA2 accumulation levels in the attached flowers from wild-type petunia plants inoculated with non-transformed *Agrobacterium* (mock control), or *Agrobacterium* bearing a TRV empty vector, TRV-*PhCHS*, or TRV-*PhCHS*/*GA20ox3* construct. Accumulation levels were standardized to *26S* *rRNA*. Error bars represent standard error of the mean from three biological replicates. Statistical significance was determined using Duncan’s multiple range test (*P* < 0.05) and shown as different letters.


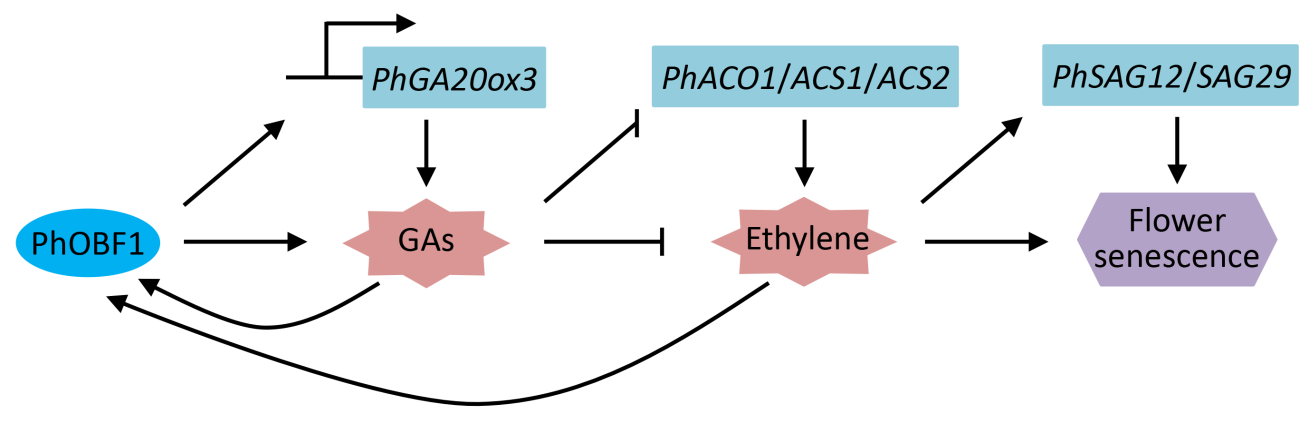


**Figure S6. A proposed model describing an important role of PhOBF1 in regulating petunia flower senescence.** PhOBF1 positively regulates the GA production by specifically binding to the promoter of GA biosynthetic gene *PhGA20ox3*. GAs have an antagonistic effect on ethylene-promoted flower senescence by reducing the transcription of ethylene biosynthetic genes *PhACO1*, *PhACS1*, and *PhACS2*. In addition, exogenous treatments with ethylene and GAs induce the expression of *PhOBF1* in petunia flowers. Solid lines ending with an arrow or a short perpendicular line represent positive or negative regulation, respectively.


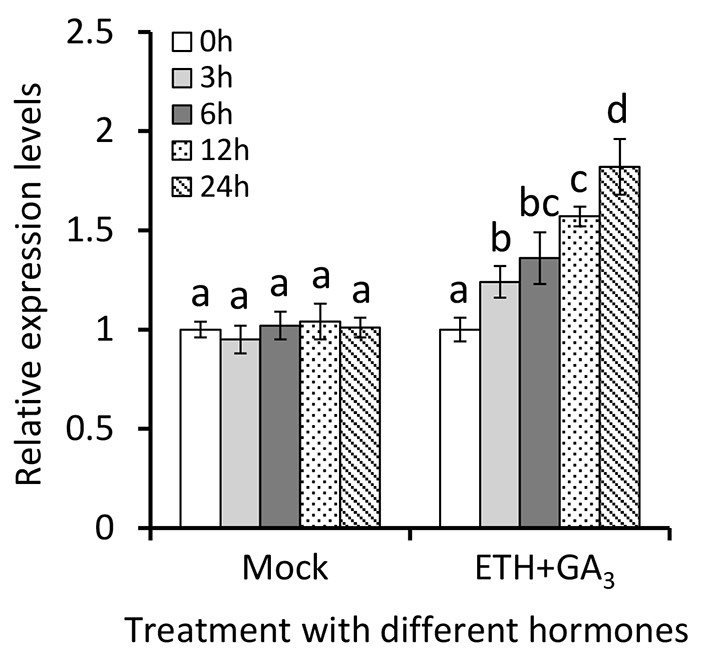


**Figure S7. Expression of *PhOBF1* in response to the combined treatment with ethylene and GA_3_.** Relative expression levels of *PhOBF1* at different hours after the co-treatment with ethylene (ETH) and GA_3_. The treatment with water was used as the control (mock). Expression levels were standardized to *26S rRNA*. Error bars represent standard error of the mean from three biological replicates. Different letters indicate statistical significance as calculated by Duncan’s multiple range test at *P* < 0.05.

**Table S1.** **Primers used for gene expression analysis and plasmid construct.**

| **Gene ID** | **Accession No.** | **Forward primer (5’-3’)** | **Reverse primer (5’-3’)** | **Product size** |
| --- | --- | --- | --- | --- |
| **For gene expression analysis** | | | | |
| *PhOBF1* | Peaxi162Scf00285g00011 | AACATGACTACAACACAATATGC | GATAGAACATATCAGCTGAAGCC | 268bp |
| *PhSAG12* | Peaxi162Scf01050g00224 | CTCTTGGACACTGCCTTTACATT | AAACACCACTGGAATAGAACTGG | 250bp |
| *PhSAG29* | Peaxi162Scf00067g00107 | ACTTCTGAGTTGGCTTTCGTCTT | TGGTGGCAAATATCAAGAAGATT | 274bp |
| RNA1 | AF406990 | TAATTGTGGATGGTTTGTCTGTG | GTCCCAAATTCTCTGTCCTCTTT | 231bp |
| RNA2 | AF406991 | GGTTACTAGCGGCACTGAATAGA | TAGTACTCCCTTGGTTCGTCGTA | 225bp |
| *PhGA20ox1* | Peaxi162Scf00988g00019 | TGCTTGCATAGAGCAGTTGTAAA | GAGTTCTCTTGTCAGCCCTGTAA | 196bp |
| *PhGA20ox2* | Peaxi162Scf00132g00116 | CTTGTTAATGACGCATGTTCAAA | ATAACCACAGTGCTCACCAATCT | 165bp |
| *PhGA20ox3* | Peaxi162Scf00000g00426 | GCTACTTCAATCACCTGAAATGG | GGATTCCTATCCCAACAATCTTC | 263bp |
| *PhGA20ox4* | Peaxi162Scf01178g00015 | GAGTCCACAAAGCAGCTACACTT | GGCAGAATATGGAAATGAGAGTG | 257bp |
| *PhGA3ox1* | Peaxi162Scf00015g00525 | TCACTAGGAATAACCAAGGACGA | ACTACCGCTCGATGTAACACACT | 299bp |
| *PhGA3ox2* | Peaxi162Scf00207g00837 | GTCTAGCTGCACATACGGACTCT | CTGATGGCGGACCATATAAATAA | 249bp |
| *PhGA2ox1* | Peaxi162Scf00035g00149 | AAGTTCAAGAATTCAATGGCAAA | CTTACGCTCTTAAACCTCCCATT | 211bp |
| *PhGA2ox2* | Peaxi162Scf00111g00922 | GATGTTGGTTTGGTCGAATACAT | GGATTCTTGAACCTCAGTACACG | 279bp |
| *PhGA2ox3* | Peaxi162Scf00111g00920 | TGAGCACTTCTCCATAGTCAACA | CCAATCTTCTTGTTGTCATAGCC | 255bp |
| *PhGID1A* | Peaxi162Scf00434g00079 | TAAGGGCAGTAGAATCCGATGTA | CCATCGAGGTTTATACAGTTTGG | 220bp |
| *PhGID1B* | Peaxi162Scf00936g00034 | AGGCTGTTGTTGTTTCTGTGAAT | TTCTCAGATTCAGTCCTCGTTTC | 277bp |
| *PhGAI* | Peaxi162Scf00305g00129 | CGGATTTACAAGATTTACCCACA | ACGCATCAGAGTTATCAGGTTGT | 289bp |
| *PhACO1* | Peaxi162Scf00047g01927 | CATTACAAGAAGTGCATGGAACA | CATCCTGGAACAGAAGGATTATG | 274bp |
| *PhACO2* | Peaxi162Scf01333g10015 | AAATACCCAAGAAAGCCTCACTC | GCACTTCTTGTAATGTCCCTTTG | 290bp |
| *PhACS1* | Peaxi162Scf00020g00149 | TTGACTTGATTGAGGACTGGATT | TGCTGGGTAGTAAGGTGAAGGTA | 212bp |
| *PhACS2* | Peaxi162Scf00118g00149 | GTCGAGTTTCGGGTTAATTTCTT | TTCCATAGCTCAATTTCTGCTTC | 249bp |
| *PhACS3* | Peaxi162Scf00096g01846 | TCATACTTCTTAGGATGGCAGGA | TTCTGACATGAATTTCGTCATTG | 252bp |
| *PhCHS* | Peaxi162Scf00536g00092 | GCCCAAGTCTAAGATTACCCATT | CTTGTCCAACCAAACTATCCAAG | 281bp |
| *PhEXP1* | Peaxi162Scf00911g00035 | GGATCAATGCTCATGCTACTTTC | TATTTGGTGGACAGAAATTGGTT | 243bp |
| *PhCP1* | Peaxi162Scf00549g00311 | ATATAATGCCTTGGGAGAGAAGG | CAACAATTCATTTGGCCTAGAAG | 241bp |
| *26S rRNA* | AF479174 | AGCTCGTTTGATTCTGATTTCCAG | GATAGGAAGAGCCGACATCGAAG | 184bp |
| **For fragment amplification in subcellular localization assay** | | | | |
| *PhOBF1* | Peaxi162Scf00285g00011 | GGTACCAGTGAAATCCTTCGTTCTGGTTT | GGATCCCATTGAACCTGATGAATTTCCAC | 501bp |
| **For fragment amplification in RNAi and overexpression assays** | | | | |
| *PhOBF1-*RNAi | Peaxi162Scf00285g00011 | ACTAGTGGCGCGCCAGTGAAATCCTTCGTTCTGGT | GGATCCATTTAAATCATTGAACCTGATGAATTTCC | 357bp |
| *PhOBF1-*OE | Peaxi162Scf00285g00011 | CTCGAGATGGCATCTTCTAGTGGAAA | GAGCTCTCAATACTGATAGAACATAT | 501bp |
| **For fragment amplification in EMSA assay** | | | | |
| *PhOBF1-*EMSA | Peaxi162Scf00285g00011 | GAATTCATGGCATCTTCTAGTGGAAA | AAGCTTTCAATACTGATAGAACATAT | 501bp |
| *pPhGA20ox3-*WT | Peaxi162Scf00000g00426 | Bio-AATATGAATTAATTTACACGTGTATTGATTAAAAA | TTTTTAATCAATACACGTGTAAATTAATTCATATT | 35bp |
| *pPhGA20ox3-*Mutant | Peaxi162Scf00000g00426 | Bio-AATATGAATTAATTTACAGCAGTATTGATTAAAAA | TTTTTAATCAATACTGCTGTAAATTAATTCATATT | 35bp |
| **For fragment amplification in yeast one-hybrid assay** | | | | |
| *PhOBF1-*Pray | Peaxi162Scf00285g00011 | GAATTCATGGCATCTTCTAGTGGAAA | CTCGAGTCAATACTGATAGAACATAT | 501bp |
| *pPhGA20ox3-*Bait | Peaxi162Scf00000g00426 | GAATTCTACCCCATGTGAATGTTGGATAC | GAGCTCCAGTCACAGCAATATTATTTTCT | 505bp |
| **For fragment amplification in dual luciferase assay** | | | | |
| *PhOBF1*-Effector | Peaxi162Scf00285g00011 | GAATTCATGGCATCTTCTAGTGGAAA | GGTACCTCAATACTGATAGAACATAT | 501bp |
| *pPhGA20ox3-*Reporter | Peaxi162Scf00000g00426 | GTCGACAACTGCAAAAATTGATGAACATT | GGATCCTGTAAGAAAATAATATTGCTGTG | 1,512bp |
| **For fragment amplification in VIGS assay** | | | | |
| *PhCHS-*VIGS | Peaxi162Scf00536g00092 | TCTAGAACCATTGGGCATTTCTG | GAATTCAGCCTTTCTCATTTCATCC | 206bp |
| *PhGA20ox3-*VIGS | Peaxi162Scf00000g00426 | GAGCTCCATGTTATGGTGAAAGCAAGTGA | CTCGAGCCATTTCAGGTGATTGAAGTAGC | 256bp |
